# HAT3-mediated acetylation of PCNA precedes PCNA monoubiquitination following exposure to UV radiation in *Leishmania donovani*

**DOI:** 10.1093/nar/gkv431

**Published:** 2015-05-06

**Authors:** Devanand Kumar, Swati Saha

**Affiliations:** Department of Microbiology, University of Delhi South Campus, Benito Juarez Road, New Delhi 110021, India

## Abstract

Histone modifications impact various processes. In examining histone acetyltranferase HAT3 of *Leishmania donovani*, we find elimination of HAT3 causes decreased cell viability due to defects in histone deposition, and aberrant cell cycle progression pattern. HAT3 associates with proliferating cell nuclear antigen (PCNA), helping load PCNA onto chromatin in proliferating cells. HAT3-nulls show heightened sensitivity to UV radiation. Following UV exposure, PCNA cycles off/on chromatin only in cells expressing HAT3. Inhibition of the ubiquitin-proteasome pathway prior to UV exposure allows accumulation of chromatin-bound PCNA, and reveals that HAT3-nulls are deficient in PCNA monoubiquitination as well as polyubiquitination. While poor monoubiquitination of PCNA may adversely affect translesion DNA synthesis-based repair processes, polyubiquitination deficiencies may result in continued retention of chromatin-bound PCNA, leading to genomic instability. On suppressing the proteasome pathway we also find that HAT3 mediates PCNA acetylation in response to UV. HAT3-mediated PCNA acetylation may serve as a flag for PCNA ubiquitination, thus aiding DNA repair. While PCNA acetylation has previously been linked to its degradation following UV exposure, this is the first report linking a HAT-mediated PCNA acetylation to PCNA monoubiquitination. These findings add a new dimension to our knowledge of the mechanisms regulating PCNA ubiquitination post-UV exposure in eukaryotes.

## INTRODUCTION

Histone modifications (reviewed in (1–4)) were first reported in the 1960s, when Allfrey *et al*. (5) showed histones to be acetylated and methylated in nuclei. Over a 100 histone residues have been found to carry post-translational modifications (PTMs) since then. Histone (PTMs) include methylation, phosphorylation, acetylation, sumoylation, ubiquitylation (reviewed in (2)) and more recently identified, hydroxylation, crotonylation, succinylation and malonylation (6,7). These PTMs attenuate the interactions of histones with DNA, thus altering local chromatin environment. The PTM marks are also a part of the ‘histone code’ that regulates various cell processes through the binding of effectors, which recognize the marks with the help of specific domains they carry (8). The effector proteins may then harness other proteins to chromatin, or may influence the modification of other histone residues. A host of histone modifying enzymes ‘write’ the ‘histone code’.

The acetylation status of histones are regulated by histone acetyltransferases (HATs) and histone deacetylases (HDACs). While the HATs help chromatin to achieve a more ‘open’ structure by acetylating the amino groups of lysine side-chains, the HDACs are associated with chromatin repression as deacetylation of these lysine residues strengthens histone-DNA interactions. Of the two types of HATs (Type A and Type B), acetylations mediated by Type B HATs, occurring prior to chromatin assembly, play a crucial role in histone deposition on chromatin; acetylations mediated by Type A HATs mostly occur on histones after they have been assembled into chromatin, and regulate various aspects of DNA metabolism. Type A HATs broadly fall into three families – the GNAT, MYST and CBP/p300 families. Of these, the CBP/p300 family has thus far been identified only in metazoans. The favored sites of acetylation are the N-terminal tails of histones, though internal sites of acetylation have also been identified.

Trypanosomatids cause diseases such as sleeping sickness, Chagas disease and Leishmaniasis. Each year 500 000 new cases of the potentially fatal Visceral Leishmaniasis (VL) are reported, 90% of which are from Sudan and the Indian subcontinent (www.who.int/emc), with *Leishmania donovani* being the causative agent of VL in India. Limited drugs are available for treatment, and hence the search for sites for therapeutic intervention continues. Understanding the effects of histone modifications is an important aspect of dissecting the cellular processes of *Leishmania*. Trypanosomatids have branched early from the eukaryotic lineage, and the sequences of trypanosomatid histones (and the accompanying PTMs) are divergent from those of other eukaryotes (9–12), with some synonymous modifications having been identified. In general, the number of histone PTMs and the enzymes that cause them are fewer than in other eukaryotes, reducing the likelihood of functional redundancies. This makes it relatively simpler to analyze the roles of individual histone PTMs and the enzymes that cause them, thus making this family of organisms a useful model system to study particular effects. The MYST family of HATs (named after the first members to be identified – **M**OZ, **Y**bf2/Sas3, **S**as2 and **T**IP60) are conserved across eukaryotes from yeast to humans (13), and also include protozoan members (14–17). Four MYST family HATs have been annotated in *Leishmania* species (11,12). This report presents the results of our efforts to characterize the *Leishmania donovani* histone acetyltransferase HAT3. Our results implicate HAT3 in modulating cell cycle and DNA repair events.

## MATERIALS AND METHODS

### Cultures, cell synchronization and flow cytometry analysis

*Leishmania donovani* 1S promastigotes were cultured as described in (18). For the analysis of growth patterns and cell viability promastigote cultures were initiated in triplicates at 1 × 10^6^ cells/ml from stationary phase cultures, aliquots withdrawn every 24 h, viable and dead cells scored and the averages of the triplicate values determined. Cell viability was determined using trypan blue exclusion method. For this, 1 μl of 0.4% trypan blue was added to 500 μl cell suspension in 1× PBS, and cells visualized immediately under a light microscope at 40× magnification. Viable cells excluded the dye. The percent cell survival was determined by dividing the number of viable cells by the total number of cells, and multiplying the value obtained by 100. For analysis of growth after exposure to UV radiation, logarithmically growing cells (Day 3 cultures; 6 × 10^5^ /ml) were exposed to UV (254 nm; using a UV torch of intensity 400 μW/cm^2^), and allowed to recover under white light after addition of equivalent amount of fresh M199 medium. *Leishmania* procyclics and metacyclics were separated as described in (19). Whole cell extracts were prepared using the M-PER kit (Pierce Biotechnologies). *Leishmania donovani* cultures were synchronized and flow cytometry analysis performed as described in (20).

### Transfections and creation of clonal lines

*Leishmania* promastigotes were transfected as described in (21). Drug was added 30–42 h after transfection for polyclonal transfectant cultures. Expression of HAT3-FLAG proteins were analyzed 8–12 days after application of drug-induced selection pressure (G418 at 100 μg/ml). For HAT assays HAT3-FLAG proteins were pulled down 2 weeks (or later) after transfections. To make clonal lines, cell clumps were removed by low-speed centrifugation (200*× g* for 4 min) 24 h after transfection, and the rest of the cells were plated on M199 semisolid medium containing the drug (G418 at 50 μg/ml, hygromycin at 16 μg/ml and bleomycin at 2.5 μg/ml). After incubation at 26°C for 10–14 days, colonies obtained were inoculated into medium containing the selection drug and gradually expanded from 1 ml to 10 ml cultures. Clonals were maintained in the presence of the specific selection drug(s).

### HAT assay

Whole cell lysates of cells expressing HAT3-FLAG proteins were incubated for 2 h at 4°C with FLAG M2-agarose beads (Sigma Aldrich, USA) equilibrated with 1× PBS. After washing the beads extensively to remove unbound/nonspecifically bound proteins, the bead-bound FLAG-tagged proteins were directly used in assays. Assays using HAT3-FLAG and HAT3-C149A-FLAG proteins were performed using HAT Assay Kit as described (Active Motif, USA; (15)), with peptide substrates (Peptron Inc, South Korea, or Abgent, USA) derived from the tails of *L. donovani* histones. Briefly, pulldowns of HAT3-FLAG and HAT3-C149A-FLAG were divided into five or six parts equivalent to ∼2 × 10^9^ cells each. One part was used to perform the assay in absence of substrate to determine autoacetylation levels, and other parts were used to perform the assay in presence of the peptide substrates to determine autoacetylation plus histone peptide acetylation levels. Each experiment was performed thrice. Mean values are depicted, with error bars showing standard deviation.

### Immunoprecipitations

PCNA immunoprecipitates were obtained from whole cell extracts of cells expressing HAT3-FLAG, by immobilizing anti-PCNA antibodies and exposing the extracts to the immobilized antibodies. For this, 10 μl rabbit PCNA antibodies (22) were incubated on ice with a 1:1 (v/v) mix of Protein A sepharose /CL6B sepharose beads (Sigma Aldrich, USA) equilibrated with 1× PBS, for 1 h with intermittent mixing. Unbound antibody was washed off with 1× PBS-0.2% Triton X-100. Lysates prepared from around 4–8 × 10^9^ cells expressing HAT3-FLAG were treated with 40 units of DNase I (New England Biolabs) for 15 min at room temperature, added to the immobilized antibodies and incubated overnight at 4°C with mixing using a nutator. The beads were washed extensively with 1× PBS-0.2% Triton X-100, boiled in SDS-PAGE sample loading buffer, released proteins resolved on SDS-PAGE and analyzed by western blot. HAT3-FLAG immunoprecipitates were similarly analyzed, using FLAG-M2 agarose beads (Sigma Aldrich, USA) to immunoprecipitate HAT3-FLAG.

### Creation of HAT3 knockout

Knockout plasmids were constructed using vectors pUC18 (NEB), pLEXSY-I-neo3 (Jena Biosciences, Germany) and pLew90 (23)as described in Supplementary Methods. In the first step, one allele was knocked out by transfecting BamHI-linearized pUC/*HAT3-KO/hyg* to create HAT3-hKO (HAT3 **h**eterozygous **k**nock**o**ut) mutant. Clonal lines were made by selection using hygromycin (16 μg/ml). HAT3 null mutants (HAT3-KO) were made by transfecting the HAT3-hKO mutant with the HAT3-KO cassette released from pLEXSY-neo/*HAT3-KO* plasmid by EcoRI-EcoRV digestion. Clonal lines were selected for using G418 (50 μg/ml) and hygromycin (16 μg/ml). To rescue the phenotype of the HAT3-null mutant, the HAT3-KO strain was transfected with plasmid pXG(*bleo*)/HAT3-FLAG and clonal lines selected using hygromycin (16 μg/ml), G418 (50 μg/ml) and bleomycin (2.5 μg/ml). A line that expressed HAT3-FLAG robustly was selected for further analysis.

### Analysis of histone acetylation after UV irradiation

Exponentially growing promastigotes (at density 1.8 × 10^6^ cells/ml) were exposed to UV radiation (254 nm; with a UV torch of intensity 400 μW/cm^2^) in a 24-well cluster dish, and allowed to recover under white light after addition of equivalent amount of fresh M199 medium. Cells were harvested at various time intervals into recovery (7.2 × 10^6^ cells per time-point), washed with 1× PBS and directly lysed in SDS-PAGE sample loading buffer. About 3.6 × 10^5^ cell equivalents of each time-point were analyzed by western blotting.

### Isolation and analysis of DNA-associated proteins

Chromatin*-*bound fraction was isolated from 5 × 10^7^ cells in the exponential phase of growth, as described in (24). Briefly, cells were harvested, washed with 1× PBS and cell pellet extracted by resuspension in detergent-containing buffer (10 mM Tris.Cl (pH 7.4), 100 mM NaCl, 300 mM sucrose, 3 mM MgCl_2_, 0.1% Triton X-100, 50 mM sodium butyrate and protease inhibitors) using a nutator, for 10 min at 4°C. Pellet was collected by low-speed centrifugation (1300 × *g*/4 min/4°C), and the supernatant (S1; soluble protein 1) was further clarified by centrifugation (20 000 ×*g*/15 min/4°C). The cell pellet from the extraction was subjected to a second round of extraction using the same lysis buffer, and the supernatant obtained (S2; soluble protein 2) was clarified by centrifugation. To release proteins associated with DNA, the pellet obtained after two rounds of extraction was subjected to DNase I treatment for 30 min at room temperature (50 units for 5 × 10^7^ cells in 60 mM Tris.Cl (pH 7.5), 2.5 mM MgCl_2_, 11 mM CaCl_2_), and the reaction centrifuged (1700 × *g*/5 min/ 4°C) to obtain supernatant (S3; DNA-associated proteins 1). The pellet obtained after centrifugation was subjected to a second round of DNase I treatment, and the supernatant saved (S4; DNA-associated proteins 2). S1–S4 fractions isolated from ∼7.5 × 10^6^ cell equivalents were analyzed by western blotting. For analysis after UV irradiation, promastigotes (Day 3 cultures; 5 × 10^7^ cells at density 1.8 × 10^6^ cells/ml) were exposed to UV radiation as described above and allowed to recover under white light after addition of equivalent amount of fresh M199 medium, before harvesting for analyses.

## RESULTS

### HAT3 is nuclear throughout the cell cycle

**Figure 1.**
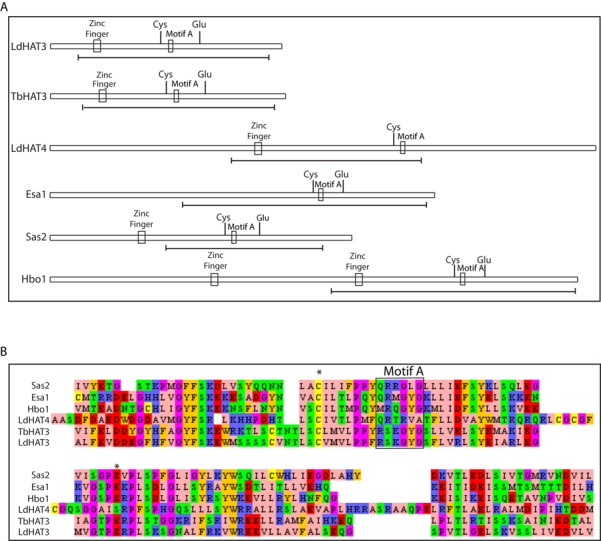
Comparison of *Leishmania donovani* HAT3 with other HATs. (**A**) Conserved domains and catalytic residues in MYST-family HATs. Core acetyltranferase domain demarcated with a line under each HAT. (**B**) ClustalW comparative analysis of LdHAT3 with other HATs viewed using Jalview multiple alignment editor (25). Asterisks mark catalytic cysteine and glutamate residues. Sas2 and Esa1 are from *Saccharomyces cerevisiae* while Hbo1 is from human cells.

The HAT3 gene was amplified from *Leishmania* genomic DNA as described in Supplementary Methods. Clones from two independent PCRs were sequenced (GenBank Accession No. KF413615). Using ClustalW analysis (25). LdHAT3 was found to share ∼38–46% identity and ∼51–62% similarity with HAT3 of *Trypanosoma* species, and 78–100% identity and 85–97% similarity with HAT3 of other *Leishmania* species. The LdHAT3 sequence showed 100% identity with HAT3 from *Leishmania donovani* strain BPK282A1 as well as *Leishmania infantum*. Based on derived amino acid sequence, the protein was found to contain all the domains and motifs that typify members of the MYST family of HATs (Figure 1). The core catalytic acetyltransferase domain (residues 32–274) carries a zinc finger motif important for substrate recognition as it is believed to interact with the globular region of the nucleosomal core (CXXCX_12_HX_3–5_C; residues 36–56); and Motif A which mediates the binding of Coenzyme A (amino acids 157–162). Analysis of the crystal structure of Esa1, a yeast HAT, has revealed that histone acetylation by MYST family members is mediated through an acetyl-Cys enzyme catalytic intermediate, the formation of which is arbitrated by a glutamate residue which is believed to be required for deprotonation of the Cys residue before acetyl group transfer (26,27). Unlike LdHAT4 (Figure 1; (15)), LdHAT3 resembles the conventional MYST family HATs such as Sas2, Esa1 and HBO1, possessing both the catalytically crucial residues (Cys and Glu) at positions 149 and 183, respectively.

**Figure 2.**
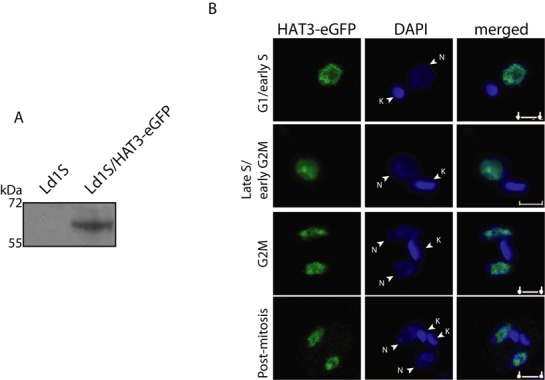
HAT3-eGFP expression. (**A**) HAT3-eGFP was immunoprecipitated from extracts of 2 × 10^8^ cells using anti-GFP antibodies (Invitrogen). Immunoprecipitates were analyzed by western blotting using anti-GFP antibody (1:1000 dilution). (**B**) Immunofluorescence analysis of HAT3-eGFP expression. G1/early S phase cells – one nucleus, one short kinetoplast. Late S phase/early G2/M cells – one nucleus, one elongated kinetoplast. G2/M phase cells – two nuclei, one kinetoplast. Postmitotic cells - two nuclei, two kinetoplasts. Cells were viewed and images acquired with a 100× objective, utilizing a confocal microscope (Leica TCS SP5) equipped with a high-resolution camera. DAPI staining indicates DNA compartments. Magnification bar represents 2 μm. N-nucleus, K-kinetoplast.

Most eukaryotic members of the MYST family of HATs are nuclear in nature. However, protozoan members have displayed variations in their distribution patterns within the cell. As nuclear localization signals are not well defined in *Leishmania* species, sequence analysis of HAT3 did not allow us to predict the subcellular localization of the protein. To examine the subcellular localization of HAT3 we took the approach of tagging the endogenously expressed protein with eGFP at its C-terminus and following it microscopically by immunofluorescence, using kinetoplast morphology and segregation pattern as cell cycle stage marker (16,20,28). Endogenous HAT3 was tagged with eGFP as described in Supplementary Methods, and the authenticity of the replacement was verified by PCRs using appropriate primers (Supplementary Figure S1A). Expression was too weak to detect in western blot analysis of whole cell extracts using the anti-GFP antibodies, and therefore, we immunoprecipitated HAT3-eGFP using the anti-GFP antibodies and analyzed the immunoprecipitates by western blot. This showed that HAT3-eGFP protein was expressed (Figure 2A). Images of direct fluorescence of HAT3-eGFP could not be captured as expression was too weak, and therefore, indirect immunofluorescence analysis was carried out using the anti-GFP antibodies to amplify the signal, as described in Supplementary Methods. This revealed that the protein remained nuclear throughout the cell cycle (Figure 2B). No immunofluorescence was detected in cells not expressing HAT3-eGFP, and treatment with tubulin antibody labeled the entire cell, confirming the specificity of the GFP antibody (Supplementary Figure S1B). Thus, HAT3 behaves like conventional MYST family HATs of yeast and higher eukaryotes, as does the *Trypanosoma brucei* HAT3 (14).

### HAT3 acetylates the 4th lysine of histone H4

**Figure 3.**
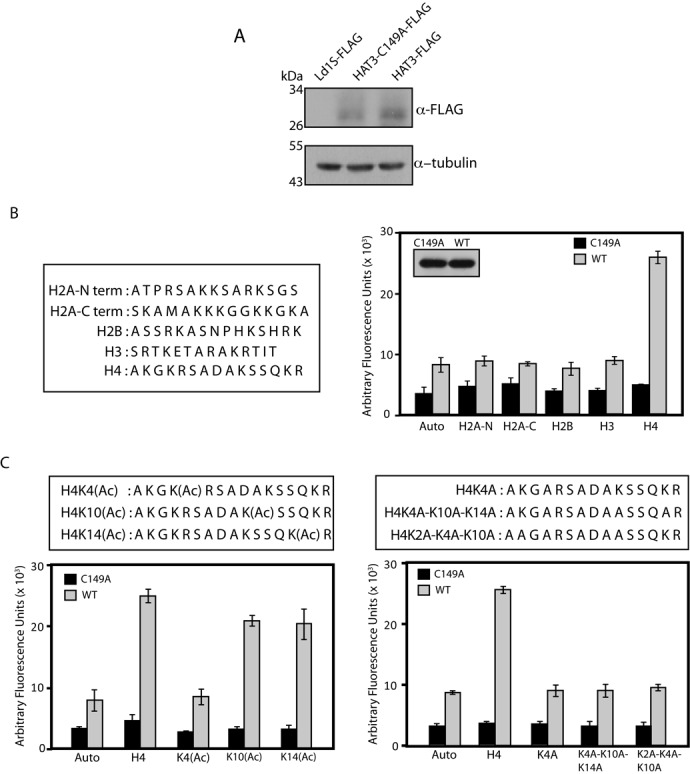
(**A**) Analysis of HAT3-FLAG expression. Western blot analysis of whole cell extracts made from 2.5 × 10^7^*Leishmania donovani* promastigotes transfected with Flag vector and from the same number of transfectants expressing HAT3-Flag and HAT3-C149-A-Flag, using anti-FLAG antibody (1:5000 dilution; Sigma Aldrich). (**B**) HAT assays using H2A, H2B, H3 and H4 peptides as substrates. HAT3-FLAG proteins (wild type and mutant) were pulled down from 1.2 × 10^10^ promastigotes. Left panel: sequences of peptide substrates. Right panel: HAT assay products were estimated after centrifugation to separate out the bead-bound protein. Inset shows western blot analysis of one-twelfth of input bead-bound proteins using anti-FLAG antibody. (**C**) HAT assay with modified H4 peptide substrates. Sequences of H4 peptide derivatives used in HAT assays are shown in the boxes. For each set of assays, HAT3-FLAG proteins were pulled down from 1 × 10^10^ promastigotes. Left panel: assays using acetylated H4 peptides as substrates. Right panel: assays using H4 peptides with altered sequence as substrates.

The substrate specificity of HAT3 was examined in a fluorescence-based assay, performed with HAT3 expressed in fusion with FLAG tag in *Leishmania* promastigotes. The assay is based on the principle that the transfer of acetyl group from acetyl CoA to histone substrate is accompanied by the generation of free Coenzyme A. The sulfhydryl groups on the free CoA are estimated. Accordingly, plasmid pXG/HAT3-FLAG was transfected into promastigotes and transfectants selected for using G418 (100 μg/ml). The HAT3 mutant HAT3-C149A (C149 being the residue predicted to be essential for catalysis, Figure 1; mutant created as described in Supplementary Methods) was similarly expressed. Expression of HAT3-FLAG and HAT3-C149A-FLAG was confirmed by western blot analysis of whole cell extracts using anti-FLAG antibodies (Sigma Aldrich, USA; Figure 3A). HAT3-FLAG proteins were effectively pulled down from extracts of logarithmically growing transfectants and activity was measured (as described in ‘Materials and Methods’ section). The activity was first tested on peptide substrates whose sequences were derived from the N- or C-termini of *Leishmania donovani* histones H2A, H2B, H3 and H4 (Figure 3B, left panel). HAT3-FLAG displayed autoacetylation activity in absence of peptide substrates, while HAT3-C149A-FLAG displayed activity levels slightly over background. While HAT3-FLAG activity remained near autoacetylation levels in presence of substrates derived from histone H2A, H2B and H3 tails, acetylation increased in presence of H4 peptide substrate, implicating H4 N-terminus to be a HAT3 target substrate (Figure 3B, right panel). All the peptides were acetylated using purified p300 (data not shown).

To ascertain which of the four lysine residues at the N-terminus of H4 are HAT3 targets, assays were carried out using pre-acetylated peptides. While pre-acetylation of H4 peptide at the 10th or 14th lysine residues did not impact HAT3-mediated H4 acetylation, pre-acetylation of H4 at the 4th lysine residue brought acetylation levels down to autoacetylation levels (Figure 3C, left panel). Mutating the 4th lysine residue to alanine had the same effect (H4K4A; Figure 3C, right panel). An H4 peptide carrying only a single lysine at the 2nd position (H4K4A-K10A-K14A) did not get acetylated either, ruling out K2 as a target site. HAT3-mediated histone H4 acetylation was found to be about 2.5- to 3-fold greater than HAT3 autoacetylation (Supplementary Figure S2). Taken together, these data show that HAT3 specifically targets the 4th lysine in histone H4 for acetylation *in vitro*.

### Analysis of H4K4 acetylation

**Figure 4.**
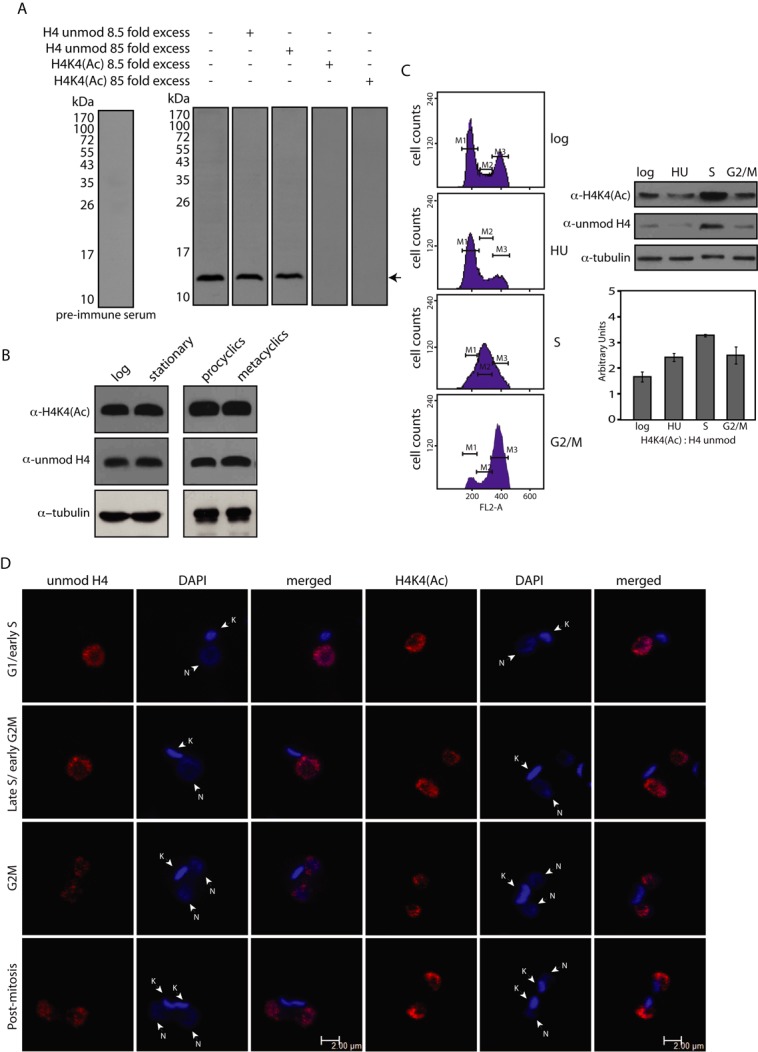
(**A**) Peptide competition assays. Antiserum pre-incubated with 8.5-fold or 85-fold excess H4 peptide (either unmodified H4 or H4K4(Ac)), before using it in western blot analyses to probe *Leishmania* extracts resolved on SDS-PAGE (15%). (**B**) H4K4 acetylation at different stages of *Leishmania*. Western blot analysis of whole cell extracts isolated from 5×10^6^ promastigotes, using anti-H4K4(Ac) antibody, anti-H4unmod antibody (both from Abgent, USA) and antitubulin antibody (Zymed, USA). (**C**) H4K4 acetylation at different stages of cell cycle. Left panel- Flow cytometry profiles of cells harvested at different times. M1, M2 and M3 indicate G1, S and G2/M phases respectively. Right panel (upper) – western blot analysis of extracts of cells harvested at each time point, using anti-H4K4(Ac), anti-H4unmod and antitubulin antibody. Right panel (lower) – ratio of H4K4(Ac): H4 unmodified at the different cell cycle stages as determined by quantification using ImageJ 1.46r software (Wayne Rasband, NIH, USA). (**D**) Subcellular localization of H4acetylK4 at different stages of the cell cycle. DAPI staining indicates DNA compartments. Magnification bar represents 2 μm. N: nucleus, K: kinetoplast.

To validate that H4K4 is a HAT3 target site *in vivo*, and to investigate the role of the H4K4 acetylation event in cellular processes, antibodies to H4acetylK4 (H4K4(Ac)) were raised in rabbit and the specificity of the antibodies analyzed using peptide competition assays in western blot analysis (as described in Supplementary Methods). Pre-incubation of the antiserum with excess unmodified H4 peptide did not impact detection of histone H4; however, pre-incubation of the antiserum with excess H4K4(Ac) peptide resulted in the modification-specific antibodies being titrated out by immunoprecipitation, due to which histone H4 was no longer detected (Figure 4A). This confirmed that the H4acetylK4 antiserum was modification-specific and did not cross-react with the unmodified H4 peptide.

The *Leishmania* parasite alternates between the insect sandfly and the mammalian host, existing in the sandfly midgut as noninfective procyclics and eventually differentiating into infective metacyclic forms that migrate to the salivary glands. *Leishmania* undergo a similar developmental pattern in axenic cultures. The acetylation of histone H4 at the 4th lysine residue was examined at different stages of the parasite's growth. We found H4K4 acetylation levels to be similar in logarithmically growing versus stationary phase promastigotes (Day 3 and Day 6 cultures respectively), and in procyclics versus metacyclics (Figure 4B). To establish the pattern of H4K4 acetylation during the different phases of the cell cycle, cells were synchronized using hydroxyurea and released into S phase. Cells harvested at various time-points were simultaneously evaluated for their DNA content (by flow cytometry) and acetylation status of H4K4. Histone synthesis generally occurs in S phase, concurrent with DNA replication. To assess H4K4 acetylation status, protein extracts were equalized for tubulin and then probed with antibodies specific to unmodified H4 peptide and H4acetylK4 peptide. We found that levels of H4 with unmodified K4 as well as acetylK4 ramped up in S phase, indicating that K4 acetylation occurs more or less concomitantly with histone synthesis in S phase (Figure 4C), although it must be kept in mind that HU treatment of cells may impact histone modifications.

In *Tetrahymena* species, *Drosophila* and mammalian cells, newly synthesized H4 is diacetylated at K5 and K12 by a type-B HAT in the cytosol prior to histone deposition (29). However, no type-B HATs have been identified in trypanosomatid genomes thus far. LdHAT3 being constitutively nuclear (Figure 2B) suggested the possibility of newly synthesized H4 being acetylated only in the nucleus after import. An alternate possibility was that some hitherto undefined cytosolic HAT could be carrying out the event *in vivo*. To explore these aspects, the distribution pattern of histone H4 with unmodified K4 and with acetylK4 was examined by western blot analysis of nuclear and cytosolic extracts isolated from logarithmically growing cells. H4 localized to the nucleus, regardless of whether it was modified at K4 or not (Supplementary Figure S3). To examine the distribution of H4acetylK4 and H4 with unmodified K4 at different phases of the cell cycle, immunofluorescence analysis was carried out. H4 was detected only in the nucleus at all stages of the cell cycle, regardless of whether the N-terminus was acetylated at K4 (Figure 4D), suggesting that H4 is imported into the nucleus very rapidly after synthesis, and the K4 acetylation event also likely occurs in the nucleus. These findings, similar to what is seen in *T. brucei* (16), suggest that while the K4 acetylation event in trypanosomatids may be synonymous with the K5 acetylation event apparent in higher eukaryotes, the patterns of the events are not identical.

### H4K4 acetylation levels are drastically downregulated in HAT3 nulls

Results from the biochemical HAT assays we performed *in vitro* (Figure 3) assigned H4K4 to be the target substrate of HAT3. To ascertain whether HAT3 acetylated this residue *in vivo* as well, and to investigate the *in vivo* relevance of the H4K4 acetylation event, a HAT3 knockout line was made as described in ‘Materials and Methods’ section. In making the HAT3-**h**eterozygous **k**nock**o**ut (HAT3-hKO), the genomic replacement was verified by amplification across the deletion junctions using appropriate primers designed as depicted in Supplementary Figure S4A. The HAT3-null line HAT3-KO was similarly analyzed by PCRs to verify its authenticity (Supplementary Figure S4B). To confirm that the line was a HAT3-null, PCRs were performed using HAT3 primers. The expected HAT3 amplicon was apparent in Ld1S but absent in HAT3-null (Supplementary Figure S4C). The HAT3 knockout was also confirmed by RT-PCR analysis using HAT3-KO and Ld1S (wild type) cells, as described in Supplementary Methods. As seen in Supplementary Figure S4D, while a healthy amount of HAT3 transcript was evident in Ld1S, no HAT3 transcript was detected in RNA isolated from HAT3-KO cells, thus verifying that we had created a HAT3-null mutant (HAT3-KO).

**Figure 5.**
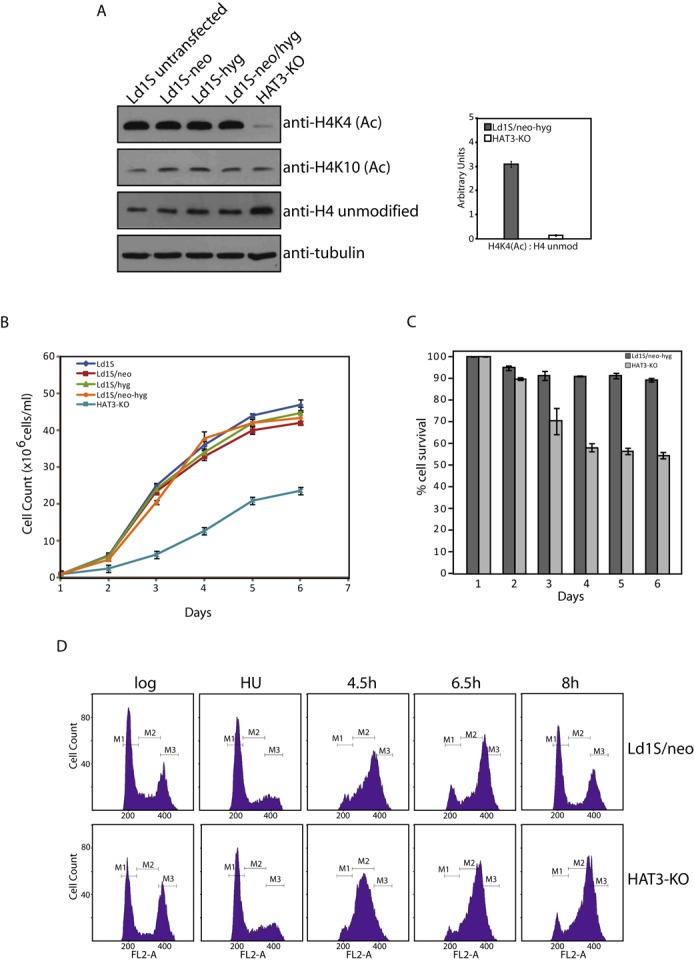
(**A**) H4K4 acetylation in HAT3-KO cells. Left panel: western blot analysis of whole cell extracts isolated from logarithmically growing HAT3-null and control promastigotes (1 × 10^6^ cell equivalents in each case), using anti-H4K4(Ac) antibody, anti-H4K10(Ac) antibody, anti-H4unmod antibody (all from Abgent, USA) and antitubulin antibody. Right panel: ratio of H4K4(Ac): H4 unmodified in Ld1S/neo-hyg and HAT3-KO cells as determined by quantification using ImageJ 1.46r software (Wayne Rasband, NIH, USA). (**B**) Growth analysis. Growth pattern of HAT3-KO cells in comparison with wild type and various control lines. (**C**) Analysis of percent cell survival of HAT3-KO cells in comparison with Ld1S/neo-hyg (**D**) Cell cycle progression. Comparison of flow cytometry profiles of HAT3-KO cells with *Leishmania donovani* cells expressing the *neo*^r^ casette. M1, M2 and M3 indicate G1, S and G2/M phases respectively.

The acetylation levels of H4 at the K4 position in HAT3-KO cells was analyzed using the acetyllysine-specific H4K4(Ac) antibodies. When extracts equalized for tubulin were probed, we found that H4K4 acetylation was largely downregulated in the null line (Figure 5A, left panel). While the ratio of H4acetylK4: unmodified H4 was ∼3 in case of wild-type cells, it was less than 0.2 in case of HAT3-KO cells (Figure 5A, right panel). In contrast, H4K10 acetylation levels remained unaffected. This was not due to the incorporation of the drug resistance cassettes as H4K4 acetylation was not affected in control lines carrying the drug markers. The low levels of H4K4 acetylation still detected in the HAT3-KO line imply that other than HAT3, some other HAT also targets H4K4 for acetylation, though it does not acetylate it as efficiently. These data unequivocally demonstrate that HAT3 is the main mediator of H4K4 acetylation *in vivo*. However, as it is not the sole mediator, it is difficult to predict if this acetylation event is essential for the cell. The subcellular localization of the residual H4K4 acetylation in HAT3-KO cells was examined microscopically and was found to be nuclear in nature (Supplementary Figure S5A), suggesting that any other enzyme responsible for this mark is also likely to be nuclear in nature.

### HAT3 modulates cell growth and cell cycle progression

The growth pattern of HAT3-KO cells was compared with that of cells expressing HAT3. A marked decrease in growth rate was apparent in cells devoid of HAT3 (Figure 5B). To ascertain if this was due to the lengthening of the generation time or/and due to decrease in cell viability, the percent of surviving cells was determined every 24 h after initiation of growth, as described in ‘Materials and Methods’ section. This analysis revealed that HAT3-KO cells showed decreased cell viability compared to cells expressing HAT3 (Figure 5C). The possibility of HAT3 modulating cell cycle progression was investigated by analyzing the HAT3-KO line in comparison with cells expressing HAT3. This was done by synchronizing HAT3-KO cells and monitoring their navigation through S phase by flow cytometry. To perform this analysis we first synchronized Ld1S cells, cells expressing the neomycin resistance cassette, cells expressing the hygromycin resistance cassette and cells expressing both drug resistance cassettes. The flow cytometry profiles of cells harvested at different time intervals revealed that cells expressing the hygromycin resistance cassette behaved like the Ld1S cells, while the cells expressing the neomycin resistance cassette moved through S phase somewhat slower (data not shown). Ld1S cells expressing both drug resistance cassettes behaved like cells expressing the neomycin resistance cassette. Therefore, we used the Ld1S cells expressing the neomycin resistance cassette as the control line for analyzing the cell cycle progression pattern of HAT3-KO cells.

For synchronization of cells, the drugs (hygromycin and/or G418) were withdrawn from the cells 72 h before setting up the hydroxyurea block. Cells arrested at G1/S boundary using hydroxyurea were released into complete drug-free M199 medium, and sampled at various time intervals thereafter for studying their cell cycle profile. Flow cytometry analysis revealed that while cells entered S phase upon release in case of both, Ld1S expressing the *neo*^r^ cassette (control) and HAT3-KO cells, the HAT3-KO cells moved through S phase somewhat slower than the control cells (Figure 5D). We also found that Ld1S (*neo*^r^) cells reached G2/M and thereafter entered G1, but HAT3-KO cells remained arrested at G2/M for a prolonged period before entering G1 phase (Figure 5D). These results suggest that HAT3 helps steer the cells through S and G2/M phases, an effect that may be mediated by its ability to acetylate H4K4.

**Figure 6.**
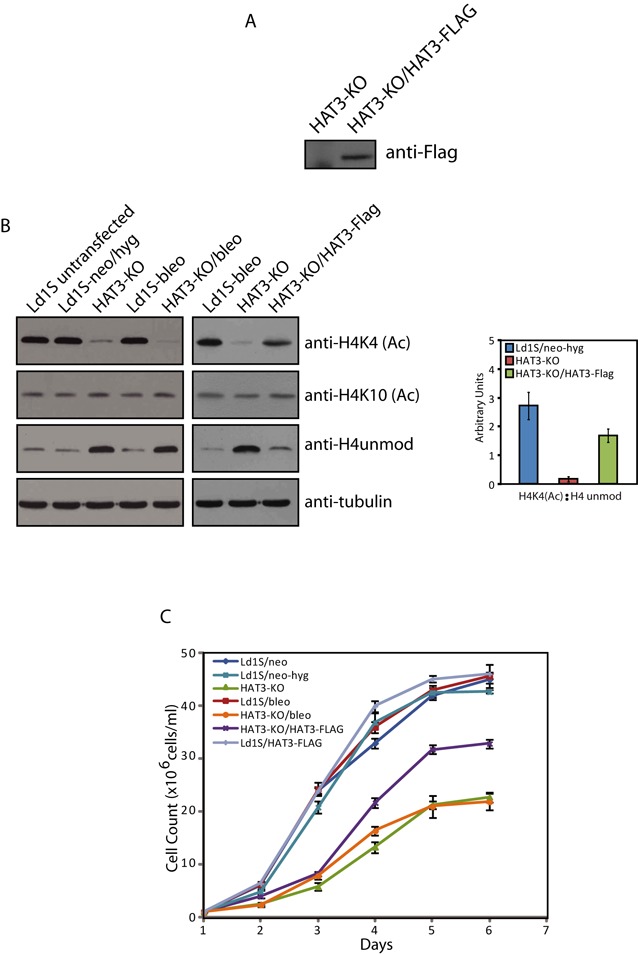
(**A**) Episomal expression of HAT3-FLAG in HAT3-KO cells. Western blot analysis of whole cell extracts made from LdHAT3-KO cells and from HAT3-KO transfectants expressing HAT3-FLAG episomally, using anti-FLAG antibody (Sigma Aldrich). (**B**) H4K4 acetylation in the HAT3-KO/HAT3 line. Left panel: western blot analysis of cell extracts of logarithmically growing HAT3-KO/HAT3 (HAT3-null/rescue line) promastigotes (1 × 10^6^ cell equivalents in each case) using anti-H4K4(Ac) antibody, anti-H4K10(Ac) antibody, anti-H4unmod antibody and antitubulin antibody. Right panel: ratio of H4K4(Ac): H4 unmodified in Ld1S/neo-hyg, HAT3-KO and HAT3-KO/HAT3 cells as determined by quantification using ImageJ 1.46r software (Wayne Rasband, NIH, USA). (**C**) Growth analysis. Growth pattern of HAT3-KO cells and HAT3-KO cells expressing HAT3-FLAG (HAT3-KO/HAT3) in comparison with wild type cells and control lines.

Attempts were made to rescue the mutant phenotype by expressing HAT3 episomally in the HAT3-KO line and analyzing its impact on H4K4 acetylation. For this, HAT3-FLAG was expressed from a plasmid carrying the bleomycin resistance gene (as described in Supplementary Methods). The plasmid pXG(*bleo*)/HAT3-FLAG was transfected into the HAT3-KO line, and one of the clones that expressed HAT3-FLAG robustly (Figure 6A) was analyzed further. A control line was also made by transfecting the pXG*(bleo)/*FLAG plasmid into promastigotes. H4K4 acetylation was analyzed in the rescue line HAT3-KO/HAT3 by western blot and, as is evident from Figure 6B (left panel), expression of HAT3 in the null line partially rescued the mutant phenotype, with elevated levels of H4K4 acetylation as compared to the null line, though not to the same extent as the wild type. While the ratio of H4acetylK4: unmodified H4 was almost 3 in case of wild-type cells, it was less than 0.2 in case of HAT3-KO cells, and ∼ 1.7 in case of the rescue line (Figure 6B, right panel). These data underline the finding that HAT3 is the main mediator of H4K4 acetylation *in vivo*. When the growth pattern of HAT3-KO cells expressing HAT3 episomally (HAT3-KO/HAT3) was compared with that of HAT3-KO cells, it was found that while cells devoid of HAT3 showed decreased growth rate, in HAT3-KO/HAT3 cells this phenotype was partially rescued (Figure 6C). A possible cause for us detecting only a partial rescue of mutant phenotype in the population could be that all cells do not continue to express HAT3-FLAG equivalently during the course of the experiment even though they originated from a single clone, and thus while some cells are completely rescued others are not rescued at all, resulting in the population as a whole showing a partial rescue phenotype. Taken together these findings suggest that HAT3 is important for cell growth and plays a role in modulating cell cycle – related events, possibly through H4K4 acetylation.

### HAT3 exists in a stable complex with PCNA in cell extracts

As H4K4 acetylation in trypanosomatids is believed to be analogous to the H4K5 acetylation event known to modulate DNA replication in other eukaryotes, the probability of delayed cell cycle progression in HAT3-KO cells being due to a negative impact on DNA replication was investigated. Toward this, the possibility of HAT3 existing as part of replisome complexes, for which PCNA has served as a molecular marker, was considered. Proliferating cell nuclear antigen (PCNA) is the highly conserved eukaryotic protein that helps clamp DNA polymerases to the DNA template during DNA synthesis. Thus, it is responsible for the processivity of DNA polymerases (reviewed in (30)). Work in *L. donovani* and *T. brucei* have shown that PCNA forms distinct nuclear foci, and localizes to active sites of DNA synthesis (22,31). Recent work in *T. brucei* has shown that depletion or overexpression of PCNA causes defects in cell proliferation, DNA replication and cell cycle progression (32).

**Figure 7.**
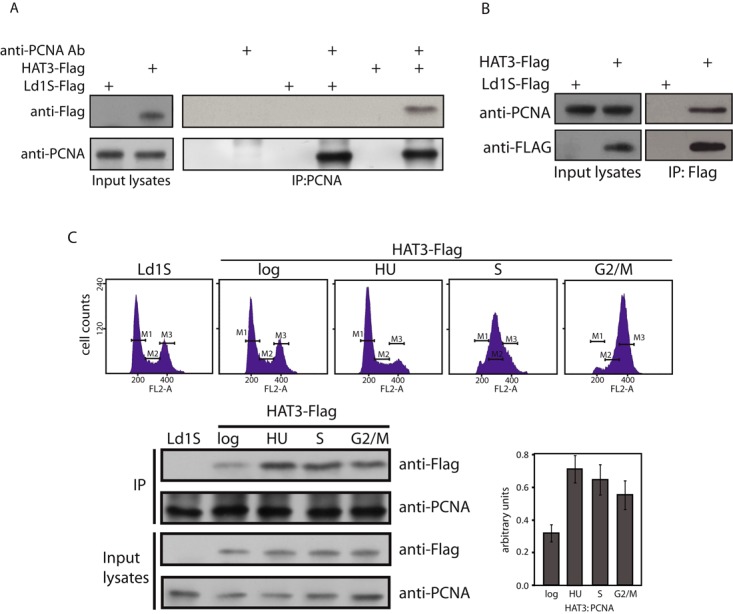
Analysis of PCNA immunoprecipitates. (**A**) PCNA was immunoprecipitated from whole cell lysates of 1 × 10^9^ logarithmically growing promastigotes. a total of 4/5 of the IP reactions were resolved by SDS-PAGE for western blot analysis. The reactions were probed with anti-PCNA and anti-FLAG antibodies. Left panels: analyses of input lysates of Ld1S transfected with empty vector (Ld1S-FLAG) or with HAT3-FLAG-expressing vector (HAT3-FLAG). Right panels: analysis of immunoprecipitation reactions. (**B**) HAT3-FLAG was immunoprecipitated from whole cell lysates of 1 × 10^9^ logarithmically growing promastigotes. A total of 4/5 of the IP reactions were similarly analyzed by western blotting. Left panels: input lysates of Ld1S transfected with empty vector (Ld1S-FLAG) or with HAT3-FLAG-expressing vector (HAT3-FLAG). Right panels: analysis of immunoprecipitation reactions. (**C**) Analysis of PCNA-HAT3-FLAG interactions in synchronized cells. PCNA immunoprecipitates from lysates of synchronized transfectant promastigotes (3.5 × 10^8^ promastigotes per time point), probed with anti-PCNA antibody or anti-FLAG antibody. HAT3-FLAG:PCNA ratio determined using ImageJ 1.46r software (Wayne Rasband, NIH, USA).

Accordingly, PCNA was immunoprecipitated from extracts of cells expressing HAT3-FLAG, and the immunoprecipitates were analyzed by western blotting using anti-FLAG antibodies. As seen in Figure 7A HAT3-FLAG co-immunoprecipitated with PCNA, indicating that it exists in a stable complex with PCNA in cell extracts. Similarly, when HAT3-FLAG immunoprecipitates from whole cell lysates were analyzed PCNA was found to co-immunoprecipitate with HAT3-FLAG (Figure 7B). The dynamics of the HAT3-PCNA complex at different stages of the cell cycle were examined by synchronizing cells expressing HAT3-FLAG and isolating whole cell lysates at the different cell cycle stages. PCNA immunoprecipitates from each of these lysates were analyzed for co-immunoprecipitating HAT3-FLAG. While equivalent levels of PCNA were immunoprecipitated from lysates isolated at the different cell cycle stages, greater amounts of HAT3-FLAG co-immunoprecipitated with PCNA from lysates of cells at the G1/S boundary, S phase and G2/M phase, than from logarithmically growing cells (Figure 7C). As about 50–60% of the log cells were in G1 and the rest in S and G2/M, this result suggests that the association of HAT3 with PCNA is more stable over S phase and G2/M.

### HAT3 may help load PCNA onto chromatin in logarithmically growing cells

**Figure 8.**
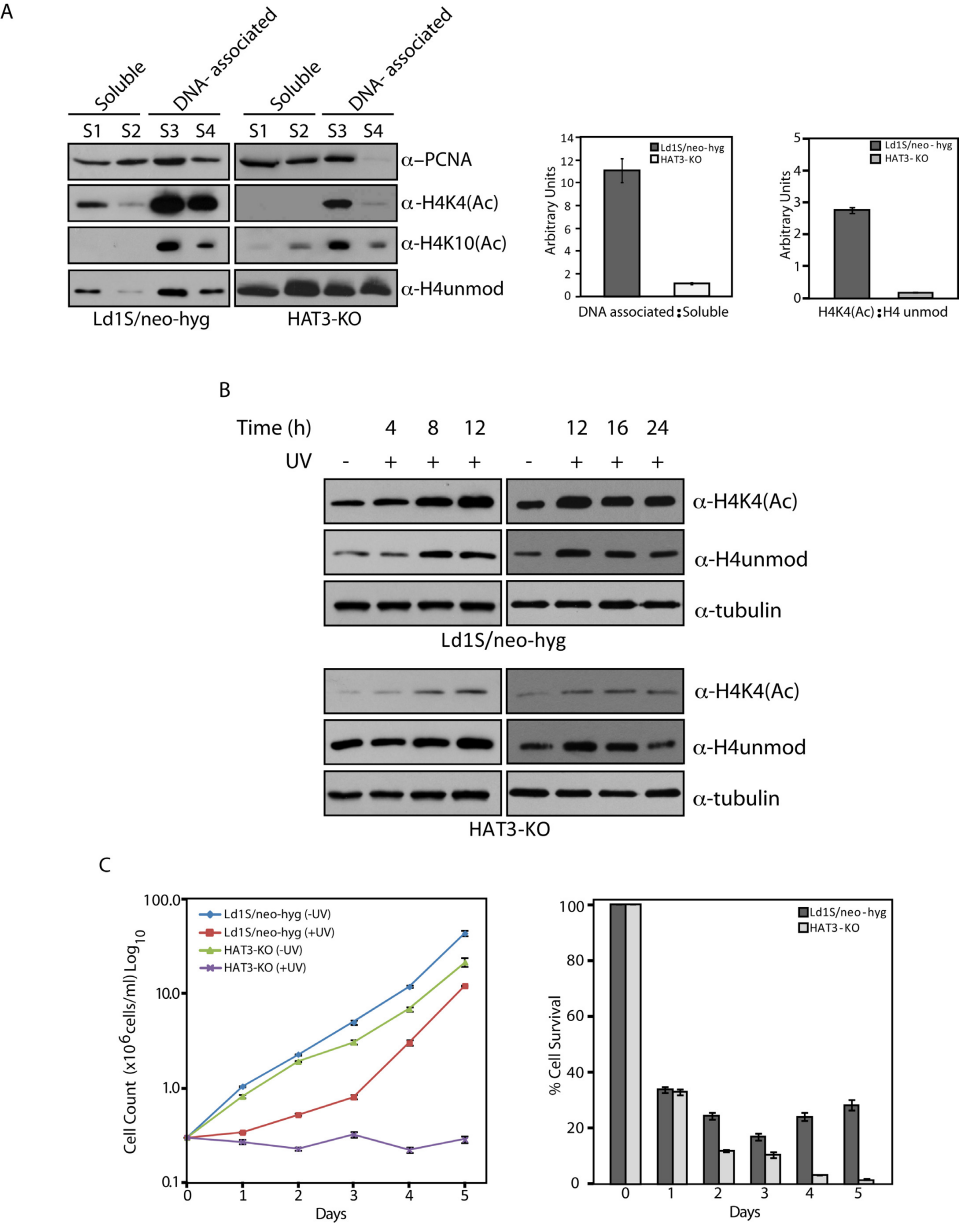
(**A**) Analysis of soluble and DNA-associated protein fractions. Left panel – fractions isolated from 7.5 × 10^6^ cell equivalents of each strain were resolved by SDS-PAGE and probed with anti-H4acetylK4 (1:1000), anti-H4acetylK10 (1:1000), anti-unmodified H4 (1:10 000) and anti-PCNA (1:5000) antibodies. S1, S2 – soluble fractions, S3, S4 – DNA-associated fractions. Center panel – ratio of DNA-associated H4 (modified + unmodified) : soluble H4 (modified + unmodified) in Ld1S/neo-hyg and HAT3-KO cells as determined by quantification using ImageJ. Right panel – ratio of total H4K4(Ac): total unmodified H4 in Ld1S/neo-hyg and HAT3-KO cells as determined by quantification using ImageJ. (**B**) Analysis of whole cell lysates at various time-points after UV irradiation. Lysates isolated from 3.5 × 10^5^ cell equivalents for each time-point were resolved by SDS-PAGE and probed with anti-H4acetylK4 (1:1000), anti-unmodified H4 (1:10 000) and antitubulin (1:5000) antibodies. (**C**) Left panel: Comparative analysis of growth of HAT3-KO cells and Ld1S/neo-hyg cells after UV irradiation. Right panel: percent cell survival of HAT3-KO cells in comparison with Ld1S/neo-hyg after UV irradiation.

Towards exploring the possible role of the interaction of HAT3 with PCNA, we isolated fractions of soluble and DNA-associated proteins from logarithmically growing cells as described in ‘Materials and Methods’ section. The fractions were analyzed for PCNA, H4acetylK4, and unmodified H4 (Figure 8A). Fractions S1 and S2 represent successively extracted soluble protein fractions while fractions S3 and S4 represent successively extracted DNA-associated proteins. The distribution of PCNA over the various fractions differed between cells merely expressing both drug resistance cassettes (Ld1S/neo-hyg) and HAT3-KO cells. A greater portion of PCNA was found in the fraction of DNA-associated proteins of Ld1S/neo-hyg than of HAT3-nulls (Figure 8A; compare S3 + S4 lanes of both cell types). This suggests that HAT3 may help recruit PCNA to chromatin in normally growing cells, an effect that might be mediated via H4K4 acetylation. To examine the possible effects of deficits in PCNA loading onto chromatin in HAT3-KO cells, on DNA replication and S phase progression, Ld1S/neo-hyg and LdHAT3-KO cells were synchronized using hydroxyurea treatment, released into S phase and analyzed for DNA replication at different time-points after release by pulsing cells with EdU and examining EdU uptake microscopically. No clear-cut difference in DNA replication pattern was evident from this (data not shown). We also analyzed Ld1S/neo-hyg and LdHAT3-KO cells that had been synchronized and released into S phase by propidium iodide staining followed by flow cytometry analysis. The number of cells in S phase at different time-points after release from HU (over 1.5 to 8 h after release) was determined using BD CellQuest Pro Software (BD Biosciences). The experiment was performed in triplicate, and mean values are presented in Supplementary Figure S5B. As apparent from the analysis, in comparison with Ld1S/neo-hyg cells, a larger proportion of LdHAT3-KO cells remain in S phase several hours after release (Supplementary Figure S5B), implying prolonged DNA replication. This is also reflected in Figure 5D.

Interestingly, the distribution of histone H4 across the various fractions (S1–S4) also differed between the two cell types. As seen in Figure 8A (left panel), while H4 was predominantly found in the fraction of DNA-associated proteins in Ld1S/neo-hyg (ratio of total H4 histones (modified plus unmodified) in the DNA-associated fractions to the total H4 histones in the soluble fractions was equal to 11; Figure 8A center panel), it was equally distributed in the fractions of soluble and DNA-associated proteins in HAT3-nulls as apparent from the western blot with antibody against unmodified H4 (ratio of total H4 histones in the DNA-associated fractions to the total H4 histones in the soluble fractions was equal to 1; Figure 8A center panel). H4 acetylated at K4, however, remained primarily chromatin-bound even in HAT3-KO cells. Thus, there is a ten-fold change in the histone distribution pattern between chromatin-bound and soluble fractions upon knockdown of H4K4 acetylation. These data suggest that H4K4 acetylation plays an important role in histone deposition into nucleosomes, with histones not acetylated on this residue remaining majorly in the soluble fraction. The deficits in histone deposition in HAT3-null cells (which could in turn lead to genomic instability) may be a primary reason for decreased viability of cells not expressing HAT3.

### HAT3-nulls show poor recovery after exposure to UV

Considering that PCNA in general plays a role in mediating not only DNA replication but DNA repair events as well, the possibility of HAT3 modulating DNA repair was addressed. The effect of UV radiation on H4K4 acetylation was examined by irradiating cells (Ld1S/neo-hyg and HAT3-nulls) and allowing them to recover for different periods of time prior to harvesting them and isolating whole cell lysates from them. As seen in Figure 8B, H4K4 acetylation increases gradually upto 12 h post irradiation, and is maintained at that level thereafter even 24 h post irradiation. Levels of unmodified H4 also increase, signifying synthesis of new histones following exposure to UV with concomitant H4K4 acetylation. To directly investigate if HAT3 plays a role in mediating cell survival after induction of DNA damage, HAT3-KO cells were insulted with UV radiation and their growth monitored in comparison with Ld1S/neo-hyg cells over 5 days. While Ld1S/neo-hyg cells gradually recovered and started multiplying HAT3-KO cells showed poor recovery (Figure 8C, left panel), with the percentage of Ld1S/neo-hyg cells that survive over time being much more than that of KO cells (Figure 8C, right panel).

### PCNA behaves dissimilarly in HAT3-nulls versus cells expressing HAT3 following exposure to UV radiation

**Figure 9.**
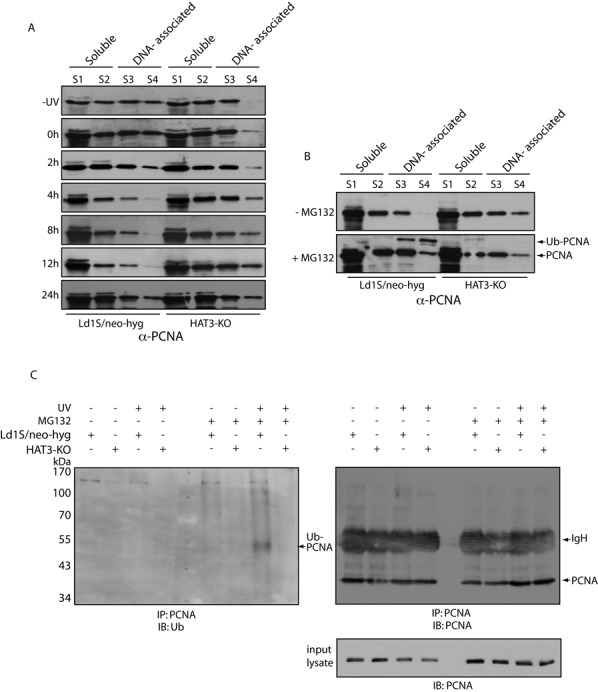
(**A**) Analysis of soluble and DNA-associated protein fractions at various time-points after exposure to UV. For each time-point, fractions isolated from 7.5 × 10^6^ cell equivalents of each strain were resolved by SDS-PAGE and probed with anti-PCNA (1:5000). S1, S2 – soluble fractions, S3, S4 – DNA-associated fractions. (**B**) Analysis of soluble and DNA-associated protein fractions 8 hours after exposure to UV. Cells were either pre-incubated with 20 μM MG132 or not. Fractions isolated from 7.5×10^6^ cell equivalents of each strain were resolved by SDS-PAGE and probed with anti-PCNA antibodies (1:5000). S1, S2 – soluble fractions, S3, S4 – DNA-associated fractions. (**C**) Analysis of PCNA immunoprecipitates either 8 h after UV or without exposure to UV. Cells were either pre-incubated with 20 μM MG132 or not. Immunoprecipitates isolated from 5 × 10^7^ cell equivalents were resolved by SDS-PAGE using a prestained molecular weight protein ladder, and analyzed by western blotting. The membrane was first probed with antiubiquitin antibody (1:1000), and the same membrane was subsequently probed with anti-PCNA antibody (1:5000). IgH-heavy chain of immunoglobulin, Ub-PCNA- ubiquitinated PCNA.

In examining how HAT3 regulates the cell's response to UV-induced DNA damage, the effect of UV radiation on PCNA distribution between soluble and DNA-associated fractions in Ld1S/neo-hyg versus HAT3-KO cells was evaluated. The association of PCNA with chromatin was found to be enhanced in HAT3-KO cells following UV irradiation (compare S4 fractions of HAT3-KO cells in –UV versus post-UV time-points in Figure 9A). In these cells, the pattern of PCNA distribution between soluble and chromatin-bound fractions remained similar in all time-points, over 2–24 h. Contrastingly, in Ld1S/neo-hyg cells, the pattern of PCNA distribution between soluble and chromatin-bound fractions varied in different time-points, over 2–24 h. PCNA cycled off/on chromatin in Ld1S/neo-hyg cells, being largely off chromatin at 8 h post UV (Figure 9A). To check if the DNA-associated fraction of PCNA in Ld1S/neo-hyg cells was being degraded during the process via the ubiquitin-proteasome pathway, cells were incubated with the proteasome inhibitor MG132 (20 μM) for 3 h prior to exposure to UV, and soluble and DNA-associated fractions analyzed 8 h after irradiation. While PCNA distribution over soluble and DNA-associated fractions in HAT3-KO cells was not affected by MG132 treatment, the protein was markedly retained on chromatin in Ld1S/neo-hyg cells treated with MG132 in comparison with untreated cells (Figure 9B). This suggested that PCNA was being degraded via the ubiquitin-proteasome pathway in Ld1S/neo-hyg cells but not HAT3-KO cells following exposure to UV. A band corresponding to the monoubiquitinated form of PCNA was also detected in the chromatin fraction (S3+S4 lanes) of Ld1S/neo-hyg cells incubated with MG132, as has been reported in other eukaryotes in response to UV. Importantly, however, monoubiquitinated PCNA was not detected in HAT3-null cells, raising the interesting possibility of the HAT3-PCNA interaction having a role additional to helping recruit PCNA to chromatin. We analyzed PCNA immunoprecipitates from untreated and MG132-treated Ld1S/neo-hyg and HAT3-KO cells that had /had not been exposed to UV radiation (lysates isolated 8 h after UV irradiation) for PCNA ubiquitination using western blot analysis with anti-Ub antibodies (Santa Cruz, USA). PCNA monoubiquitination was only detected in immunoprecipitates from Ld1S/neo-hyg cells that had been treated with MG132 prior to UV irradiation (Figure 9C). Furthermore, polyubiquitinated forms of PCNA (indicated by a streak from the mono-Ub PCNA band upwards) were also detected only in these same immunoprecipitates (Figure 9C). Cells from either strain that had not been exposed to UV or not been pretreated with MG132 did not display any PCNA ubiquitination.

Taken together, the data suggest two differences in the way Ld1S/neo-hyg and HAT3-KO cells respond to UV-induced DNA damage. First, HAT3-KO cells are deficient in PCNA monoubiquitination (Figure 9B and C), and second, PCNA is degraded via the ubiquitin-proteasome pathway in Ld1S/neo-hyg but not HAT3-KO cells (Figure 9A – C).

### The role of HAT3 in facilitating PCNA degradation following exposure to UV

**Figure 10.**
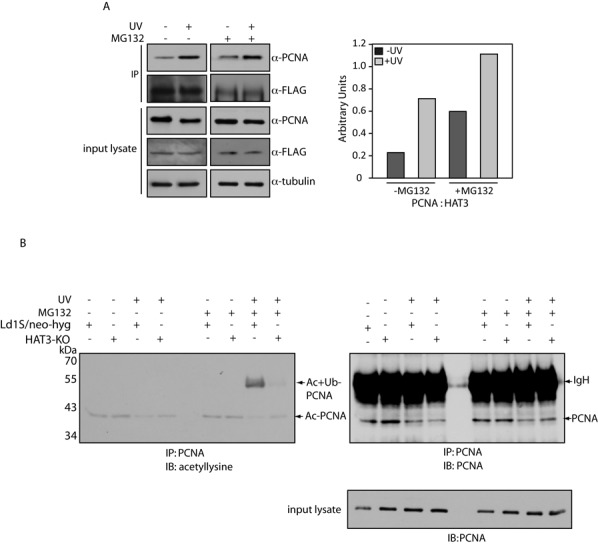
(**A**) Analysis of HAT3-FLAG immunoprecipitates either 8 h after UV or without exposure to UV. Cells were either pre-incubated with 20 μM MG132 or not. Left panel: Immunoprecipitates isolated from 1 × 10^8^ cells were probed in western blots using either anti-PCNA antibodies (1:5000) or anti-FLAG antibodies (1:1000). Right panel: PCNA:HAT3-FLAG ratio determined using ImageJ 1.46r software (Wayne Rasband, NIH, USA). (**B**) Analysis of PCNA immunoprecipitates either 8 h after UV or without exposure to UV. Cells were either pre-incubated with 20 μM MG132 or not. Immunoprecipitates isolated from 5 × 10^7^ cell equivalents were resolved by SDS-PAGE using a prestained molecular weight protein ladder, and analyzed by western blotting. The membrane was first probed with anti-acetyllysine antibody (1:2000), and the same membrane was subsequently probed with anti-PCNA antibody (1:5000). IgH-heavy chain of immunoglobulin, Ac-PCNA- acetylated PCNA, Ac+Ub-PCNA- acetylated + ubiquitinated PCNA.

In order to analyze possible roles of HAT3 in facilitating PCNA degradation by the ubiquitin-proteasome pathway following exposure to UV radiation, the HAT3-PCNA interaction in cells that had been exposed to UV was compared with the interaction in nonirradiated cells. Accordingly, cells expressing HAT3-FLAG were exposed to UV radiation, HAT3-FLAG immunoprecipitated from whole cell lysates isolated from these cells 8 h after exposure to UV, and the immunoprecipitates analyzed for co-immunoprecipitating PCNA. The amount of PCNA that co-immunoprecipitated with HAT3-FLAG was greater in case of cells that had been irradiated than in cells that had not been exposed to UV rays, and this enhanced interaction between HAT3-FLAG and PCNA was evident regardless of whether cells had been incubated with MG132 prior to irradiation (Figure 10A).

These findings raised the question of what the outcome of a heightened interaction between HAT3 and PCNA (post-UV) could be. PCNA has been reported to be acetylated in human cells post-UV exposure (33,34), and the HATs responsible for this acetylation were recently identified as the human CBP/p300 (35). To explore the possibility of HAT3 playing a role in acetylating PCNA after UV irradiation, PCNA was immunoprecipitated from UV-irradiated Ld1S/neo-hyg and HAT3-KO cells (lysates isolated 8 h after UV irradiation) and analyzed for acetylation using pan acetyllysine-specific antibodies (Abcam, USA). While basal levels of acetylated PCNA were evident in almost all cases, prominently hyperacetylated PCNA was only detected in immunoprecipitates from UV-irradiated Ld1S/neo-hyg cells that had been incubated with MG132 before irradiation (Figure 10B). This acetylated form of PCNA migrated at the same position as monoubiquitinated PCNA, suggesting the presence of an acetylated form of monoubiquitinated PCNA in Ld1S/neo-hyg cells (but not HAT3-KO cells) that had been treated with MG132 prior to UV irradiation. In absence of UV irradiation this form of PCNA was not detected. The results obtained suggest that PCNA is strongly acetylated in response to UV, and HAT3 plays a major role in mediating the acetylation of PCNA in response to UV-induced DNA damage.

## DISCUSSION

The first HAT to be identified in protozoans was the GCN5-like HAT purified from the free-living *Tetrahymena thermophila* (36,37). Since then, enzymes that mediate histone acetylations have been characterized in *Plasmodium falciparum*, *Toxoplasma gondii* (17,38–41) and trypanosomatids. The identification of histone modifications in *Trypanosoma brucei* and *Trypanosoma cruzi* has revealed that the H4 N-terminal tail carries several acetylation (and methylation) marks (42,43). The most abundant mark, found in 73% of H4, appears to be the acetylation mark on H4K4. Although specific histone modification marks have not been previously identified in *Leishmania* species, on comparing the sequences of *Leishmania* and *Trypanosoma* histones we found them to be highly conserved (15), suggesting that many of the PTMs may also be conserved. Analysis of the whole genome sequences of trypanosomatids has revealed the presence of a similar assemblage of candidate histone modifying enzymes across all of them, and the factors responsible for some of the histone PTMs have been identified in *T. brucei*, where H4K4 and H4K10 acetylation is brought about by HAT3 and HAT2, respectively (14,16), and di- and trimethylation of H3K76 is mediated by DOT1A and DOT1B methylases respectively (44). While HAT1 and HAT2 are essential to cell survival, with HAT1 also regulating the expression of genes located subtelomerically (14), DOT1A depletion abrogates DNA replication without affecting karyokinesis, with DOT1A overexpression causing continuous replication of DNA (45).

Of the four MYST family HATs identified in *Leishmania*, all are found in *T. cruzi*, while one (HAT4) is absent in *T. brucei* (11,12). HAT3 carries all the sequence motifs and catalytically vital residues that are characteristic of the members of this class of HATs (Figure 1), and we were able to abrogate HAT3 acetyltransferase activity by mutating Cys149 to alanine (Figure 3), indicating that the mechanism of action of HAT3 is similar to that of the typical MYST family HATs. *In vitro* biochemical assays revealed that LdHAT3 targets the 4th lysine residue of histone H4 (Figure 3), and analysis of cells in which HAT3 had been eliminated ascertained that HAT3 was the principal mediator of H4K4 acetylation *in vivo* (Figure 5A). Combining the observations that LdHAT3 is constitutively nuclear (Figure 2B), histone nuclear import occurs very rapidly after its synthesis (Figure 4D), and HAT3 is the primary mediator of H4K4 acetylation *in vivo* (Figure 5A), it appears that this acetylation event occurs mainly in the nucleus. HAT3 knockout negatively impacts cell viability, accentuating the importance of this HAT to cell growth and survival (Figure 5B), unlike what has been reported in *T. brucei* where knocking out HAT3 does not seem to have any effect on cell growth (14). The H4K5 acetylation event conserved across eukaryotes from yeast to humans plays a role in histone deposition (29), DNA damage repair (46), transcriptional activation (47) and cell cycle progression (48). We found that HAT3-nulls displayed aberrations in histone deposition (Figure 8A), a probable cause for decreased viability of these cells.

Exposure to DNA damage-inducing agents leads to a variety of DNA lesions, with bulky thymine-thymine dimers being formed due to UV irradiation. Eukaryotes use the highly conserved mechanism of translesion DNA synthesis to overcome the effects of such damage. This process is mediated by the TLS (**t**rans**l**esion DNA **s**ynthesis) polymerases of the Y family, which (unlike the replicative DNA polymerases) carry active sites that are designed to accomodate bulky DNA adducts, and are thus able to synthesize DNA across the site of the lesion (49,50). The TLS polymerases function in partnership with the DNA polymerase clamp protein PCNA, and the monoubiquitination of PCNA is critical to modulating the process (30,51–52). Protein ubiquitination events regulate multiple facets of protein function, like protein–protein interactions, protein degradation, localization within the cell etc. Typically, monoubiquitination (and diubiquitination) of proteins regulates processes such as DNA repair, gene expression and endocytosis, while polyubiquitination regulates DNA damage tolerance, proteasome-mediated degradation, signaling events (reviewed in (53)). Studies in *S. cerevisiae* and *Xenopus* have shown that PCNA is first monoubiquitinated (and diubiquitinated) on the highly conserved K164 following DNA damage, and subsequently polyubiquitinated (54–57). While monoubiquitination promotes TLS-based DNA repair synthesis, polyubiquitination at K63 promotes error-free damage repair through as yet poorly understood mechanisms (reviewed in (58)). Monoubiquitination in response to UV irradiation is a prominently evident PCNA modification, while polyubiquitination is detected at much lower levels (30,59–61). PCNA monoubiquitination is believed to increase the binding affinity of PCNA for TLS polymerases that carry ubiquitin-binding domains (62–65), with failure of PCNA monoubiquitination resulting in increased susceptibility to UV radiation (61). Ubiquitin-dependent bypass DNA synthesis operates mainly in S phase, and while it is error-prone as the TLS polymerases lack proof-reading ability it allows the replication fork to advance, thus protecting the genome from greater damage due to double-strand breaks resulting from replication fork collapse (reviewed in (66).

Our results point to HAT3 being a modulator of PCNA ubiquitination following exposure to UV radiation. In cells expressing HAT3 the chromatin-bound fraction of PCNA is degraded a few hours after UV irradiation, but the addition of an inhibitor of the ubiquitin-proteasome pathway allows the retention of DNA-associated PCNA in these cells. Furthermore, the monoubiquitinated form of PCNA is detected in the DNA-associated protein fraction of these cells. In stark contrast, cells not expressing HAT3 show a continued retention of chromatin-bound PCNA regardless of the presence/absence of proteasome inhibitor, and importantly, we are unable to detect the monoubiquitinated form of PCNA in these cells (Figure 9). Deficiencies in PCNA monoubiquitination in HAT3-nulls may negatively impact interactions between PCNA and TLS polymerases, thus having an adverse effect on translesion DNA synthesis-based repair processes. This would ultimately translate into decreased cell recovery following exposure to UV radiation. Thus, absence of HAT3 in cells leads to a heightened sensitivity to UV. Additionally, continued retention of PCNA on chromatin after exposure to UV in HAT3-nulls (as compared to cycling on-off chromatin in case of cells expressing HAT3) may lead to genomic instability. Coupled to the facts that HAT3 mediates PCNA acetylation, and ubiquitinated PCNA is detected in cells expressing HAT3 but not in HAT3-nulls (Figures 9 and 10), it is possible that HAT3-mediated acetylation marks PCNA for ubiquitination (mono followed by poly). This would aid DNA repair post-UV exposure via the TLS pathway, followed by PCNA turnover, which is essential for maintaining genomic stability (67).

Three HATs have been found to interact with PCNA so far. The interaction of the human p300 with PCNA is believed to assist chromatin remodeling at sites of DNA damage (68), and the interaction of yeast Elongator HAT with PCNA is proposed to facilitate DNA replication and repair (69). More recently, the interaction of CBP with PCNA has been found to play a role in mediating the cell's response to DNA damage (35). While PCNA acetylation post-UV exposure and its subsequent degradation has been reported earlier (33,34), the protein mediating this acetylation was largely unknown till recently (35). Cazzalini *et al*. have identified the HATs that acetylate PCNA in human cells (p300/CBP) and find that PCNA acetylation by these HATs is crucial to PCNA loading onto chromatin and post-repair degradation of chromatin-bound PCNA. Other than p300/CBP in human cells, the present report is the only study identifying a HAT that mediates PCNA acetylation in response to DNA damage. Importantly, this is the first report linking PCNA acetylation to PCNA monoubiquitination, a process that is believed to be critical for translesion DNA synthesis. Our data add a new dimension to what is known about the mechanisms regulating PCNA ubiquitination post-UV exposure in eukaryotes.

## ACCESSION NUMBER

KF413615.

## SUPPLEMENTARY DATA

Supplementary Data are available at NAR Online.

SUPPLEMENTARY DATA
