# Supplementary material for: HAT3-mediated acetylation of PCNA precedes PCNA monoubiquitination following exposure to UV radiation in Leishmania donovani
Source: Nucleic Acids Res. 2015 May 6;43(11):5423–41. doi: 10.1093/nar/gkv431 (PMC4477661; doi:10.1093/nar/gkv431)
Supplement: SUPPLEMENTARY DATA [file supp_43_11_5423__index.html]

HAT3-mediated acetylation of PCNA precedes PCNA monoubiquitination following exposure to UV radiation in Leishmania donovani — HAT3-mediated acetylation of PCNA precedes PCNA monoubiquitination following exposure to UV radiation in Leishmania donovani — SUPPLEMENTARY DATA 

# HAT3-mediated acetylation of PCNA precedes PCNA monoubiquitination following exposure to UV radiation in *Leishmania donovani*

## SUPPLEMENTARY DATA

- SUPPLEMENTARY DATA
