# Supplementary material for: HAT3-mediated acetylation of PCNA precedes PCNA monoubiquitination following exposure to UV radiation in Leishmania donovani
Source: Nucleic Acids Res. 2015 May 6;43(11):5423–41. doi: 10.1093/nar/gkv431 (PMC4477661; doi:10.1093/nar/gkv431)
Supplement: SUPPLEMENTARY DATA [file supp_gkv431_nar-00378-x-2015-File012.pdf]

Supplementary Methods:

Cloning of HAT3 gene

Leishmania donovani HAT3 was amplified from genomic DNA using Phu DNA polymerase (Thermo Fischer, USA) and primer pair HAT3-F and HAT3-R (5'CACCGAATTCATGAAC GTCGTTTCCGT 3' and 5'TAACCATGGTCTCCCGACGGCGCGCT 3' respectively) that were designed based on the published *Leishmania major* sequence (TriTrypDB ID: LmjF.36.6990). The ~0.8 kb amplicon obtained was cloned into pENTR/D-TOPO (Invitrogen) for sequencing. For expression in *Leishmania* promastigotes, the LdHAT3 gene was amplified using pENTR/LdHAT3 as template and primers HAT3-GFP-F and HAT3-GFP-R (5'CACCGGCCGTGCCGGCCACCTT ATGAACGTCGTTTCC 3' and 5'TGGCCGGCACGGCCTCCCGACGGCGCGCTGGT 3' respectively), and the amplicon was cloned into the SfiI site of pXG-/GFP+/FLAG (19), creating plasmid pXG/HAT3-FLAG. To create the LdHAT3-C149A mutant by overlap PCR, the N-terminal part of the mutant gene was amplified using primers HAT3-GFP-F and HAT3-C149A-R (5'GCGGCAGCACCATCAC**GGC**CGATAGCGTGTTCACA 3'), while the C-terminal part of the mutant gene was amplified using HAT3-C149A-F (5'TGTGAACAC GCTATCG**GCC**GTGATGGTGCTGCCGC 3') and HAT3-GFP-R. The full-length mutant LdHAT3-C149A gene was obtained using the mixture of the two amplicons as template, and primers HAT3-GFP-F and HAT3-GFP-R. The amplicon was cloned into pXG-/GFP+/FLAG to create the plasmid pXG/HAT3-C149A-FLAG for expression in *Leishmania*. Plasmid pXG*(bleo)/*FLAG was constructed by amplifying the bleomycin resistance cassette from pLew82 (a gift from Prof. George Cross' lab; (69)) using primers Bleo-F and Bleo-R (5'TCCTCGAGGGTTCCTGCCCATTTAGTTGG 3' and 5'GACCATGGGTCGACACACCA AGACGAGA 3' respectively), and cloning the amplicon into pXG-/GFP+/FLAG in place of the neomycin resistance cassette. The HAT3 gene was cloned into the SfiI site of pXG*(bleo)/*FLAG as described above to create the plasmid pXG*(bleo)*/HAT3-FLAG.

Creation of donor plasmid construct and tagging endogenous HAT3 with eGFP

For tagging endogenous HAT3 with eGFP, the donor plasmid was constructed using pLEXSY-I-neo3 as the backbone. The 3' Flank sequence of HAT3 was cloned into the SpeI site downstream of the *neo* marker, while the 5' Flank sequence of HAT3 along with the HAT3 gene in fusion with eGFP, replaced the XbaI-KpnI stuffer region of the vector. The plasmid created was named pLEXSY-neo/*HAT3-eGFP*. The replacement cassette carrying HAT3-eGFP (along with HAT3 flank sequences) was released from the donor plasmid using EcoRV-BglII restriction digestion, and transfected into Ld1S promastigotes. Clonal lines were selected for using G418 (50 μg/ml). Thus, one of the HAT3 genomic alleles was tagged with eGFP.

Construction of knockout plasmids

Knockout constructs were made using two backbone vectors - pUC/*hygro* made as part of this study, and pLEXSY-I-neo3 (purchased from Jena Biosciences, Germany). The hyg^r cassette was amplified using plasmid pLew90 (a gift from Prof. George Cross' laboratory; (69)) as template and primers Hyg-F and Hyg-R (5'CCGATATCATGAAAAAGCCTGAA 3' and 5'ATCCCGGGCTACTCTATTCCTTT 3'). These primers were designed such that the antibiotic resistance marker gene would be flanked by EcoRV and SmaI sites at the 5' and 3' ends respectively. The amplicon was cloned into the SmaI site of pUC19 (causing a loss of this SmaI site during the cloning process) to generate the pUC/*hygro* vector.

The 5' Flank sequence of the HAT3 gene (~1.5 kb) was amplified from genomic DNA using primers HAT3-5'FL-F and HAT3-5'FL-R (5'GAAAAACTTCTGGAGGGATGTTG 3' and 5'ACCATCACCCCCACAGCCGTC 3' respectively), and cloned into the EcoRV site of pUC/*hygro*, generating plasmid pUC/*HAT3-5'FL/hyg*. The 3' Flank sequence of the HAT3 gene (~1.5 kb) was amplified from genomic DNA using primers HAT3-3'FL-F and HAT3-3'FL-R (5'AGGATTGATGACTGTTTGTGTT 3' and 5'ATCAGGTTCTCCAGCGACGCT 3' respectively), and cloned into the SmaI site of pUC/*HAT3-5'FL/hyg*, generating HAT3 knockout

plasmid pUC/*HAT3-KO/hyg*. The second knockout construct was made using pLEXSY-I-neo3 (Jena Biosciences, Germany) as the backbone. The 3' Flank sequence was cloned into the SpeI site downstream of the *neo* marker, while the 5' Flank sequence replaced the XbaI-NotI stuffer region of the vector. The plasmid created was named pLEXSY-neo/*HAT3-KO.*

Isolation of RNA and RT-PCRs

Total RNA was prepared from $5x10^7$ logarithmically growing promastigotes using the PureLink RNA mini kit (Invitrogen, USA), according to the manufacturer's instructions. The RNA was treated with DNaseI (1 U DNase I per 2 µg RNA at 37°C for 30 min followed by the addition of 2.5 mM EDTA and incubation at 65°C for 10 min to stop the reaction) prior to cDNA synthesis. Total cDNA was synthesized from 500 ng of DNase I-treated RNA using the iScript cDNA synthesis kit (Bio-Rad Laboratories, USA) as per manufacturer's instructions. One-tenth of the synthesized cDNA was used as template per PCR reaction. For analysis of tubulin expression the primers used were Tubulin RT-F1 and Tubulin RT-R2 (5'CTTCAAGTGCGGCATCAACTA3' and 5'TTAGTACTCCTCGACGTCCTC 3' respectively). For analysis of HAT3 expression the primers used were HAT3-RT-F2 and HAT3-RT-R1 (5'GGTAGCTTTCTTGTCCGACT 3' and 5'ACCAGAACGTCTTCGACAATC 3' respectively).

Raising antibodies to acetylated histone H4 and Peptide Competition assays

Polyclonal antibodies to H4K4(Ac) peptide (and unmodified H4K4 peptide) were raised in rabbit and purified against unmodified H4 peptide using affinity-based chromatography so that only antisera to acetylated peptide were obtained, by Abgent, USA. The peptide used was derived from the H4 N-terminus (sequence: AKGKRSADAKSSQKR).

To ascertain the specificity of the antibodies to histone H4K4(Ac) sequence, peptide competition assays were carried out. Briefly, the affinity purified anti-H4K4(Ac) antibodies were incubated with unmodified or acetylatedH4 peptide (at peptide:antibody molar ratios of 8.5:1 and 85:1) in 1XPBS containing 5% BSA, and incubated with gentle mixing at 4°C overnight. The immunoprecipitates formed were removed by by high speed centrifugation and the supernatant fraction used directly as primary antibody preparation in Western Blot analysis of *Leishmania donovani* whole cell extracts.

Immunofluorescence analysis

For examining the subcellular localization of LdHAT3, promastigotes expressing HAT3-eGFP were harvested and fixed with 2% PFA, cell spreads made, the cells permeabilized using 0.1 % Triton X-100, blocked with 10% chicken serum, washed, and incubated with anti-GFP antibody (Invitrogen; Cat. No. A11122). Following washes, anti-rabbit secondary antibody labeled with FITC (Jackson Laboratories, USA; 1:100) was added. After washing off the unbound fraction, the cells were mounted in anti-fade solution containing DAPI (Vectashield, Vector Laboratories). Cells were imaged using a confocal microscope (LeicaTCS SP5) with a 100X objective. For examining the subcellular localization of H4, antibodies to unmodified H4 peptide and H4acetylK4 peptide (Abgent, USA, 1:1000 dilution) were used to probe cell spreads, followed by probing with anti-rabbit, Texas Red-labelled, secondary antibody (Jackson Laboratories, USA; 1:100) and mounting in DAPI-containing anti-fade solution.

Supplementary Figure legends:

Figure S1: A. Screening of line expressing endogenous HAT3 tagged with eGFP. PCRs performed with primer pairs whose sequences were designed from eGFP sequence, Neo sequence, and flanking *Leishmania* genome sequences. Primer pairs used are indicated above the agarose gel images, and positions of primers are indicated in the line diagram. Lane 1- Ld1S genomic DNA, lane 2- genomic DNA from Ld1S cells carrying a HAT3-eGFP allele. Arrowheads indicate PCR products of expected sizes. M- DNA ladder. **B.** upper row- immunofluorescence analysis of untransfected Ld1S cells with anti-GFP antibody (Invitrogen, USA). No green fluorescence is

detected as eGFP is absent. Lower panel- immunofluorescence analysis of HAT3-eGFP expressing cells with anti-tubulin antibody (Zymed, USA).

Figure S2A-C: Analysis of HAT3 acetylation activity using various histone peptide substrates in comparison with HAT3 autoacetylation. Bars indicate fold increase in activity over autoacetylation values when peptide substrate is added to the reaction.

Figure S3: Analysis of nuclear and cytosolic extracts of *L. donovani* for H4acetylK4 expression. Nuclear and cytosolic extracts were made using the NE-PER kit from Pierce Biotechnologies according to the manufacturer's instructions, resolved by SDS-PAGE, and subjected to Western blotting using anti-GAPDH antibodies (Sigma Aldrich), and anti-H4 (unmodified) antibodies or anti-H4K4(Ac) antibodies (both from Abgent, USA).

Figure S4: Screening of LdHAT3 knockout lines. A. Heterozygous knockout. PCRs performed with primer pairs whose sequences were designed from HYG sequence and flanking *Leishmania* genome sequences. PCRs with Orc-F and Orc-R primers served as positive control for templates. Primer pairs used are indicated above the agarose gel images, and positions of primers are indicated in the line diagram. Lane 1- Ld1S genomic DNA, lane 2- genomic DNA from LdHAT3-hoKO cells. Arrowheads indicate PCR products of expected sizes. M- DNA ladder. **B. HAT3 null line.** PCRs performed with primer pairs whose sequences were designed from UTR2 sequence, Neo sequence and flanking *Leishmania* genome sequences. Primer pairs used are indicated above the agarose gel images, and positions of primers are indicated in the line diagram. Lane 1- Ld1S genomic DNA, lane 2- genomic DNA from LdHAT3-KO cells. Arrowheads indicate PCR products of expected sizes. M- DNA ladder.**C. Confirmation of HAT3-KO (null) line.** PCRs performed with HAT3 primers using genomic DNA of Ld1S (control) and HAT3 null (HAT3-KO) as template (lanes 1 and 2 respectively). PCRs with ORC primers served as positive control. Arrowheads indicate PCR products of expected sizes. **D. RT-PCR analysis of Ld1S and LdHAT3-KO cells.** –RT: non-reverse transcribed RNA as template. +RT: reverse transcribed RNA as template. Reactions with tubulin primers served as positive control and –RT reactions as negative control.

Figure S5: A. Analysis of H4K4 acetylation in HAT3-null cells. Cells were viewed and images acquired using a100X objective, utilizing a confocal microscope (Leica TCS SP5) equipped with a high-resolution camera. DAPI staining indicates DNA compartments. Magnification bar represents 2 μm. **B. Analysis of number of cells in S phase in Ld1S/neo-hyg versus HAT3-nulls after synchronization and release.** Cells of both types were synchronized using HU, released into S phase, and harvested at 1.5 h, 3 h, 4.5 h, 6.5 h and 8 h after release. Harvested cells were stained with propidium iodide and analyzed by flow cytometry. The number of cells in S phase at each time-point was quantitated using BD CellQuest Pro Software (BD Biosciences). The experiment was performed thrice and mean values are presented in the form of a bar graph.

Figure S1

A.

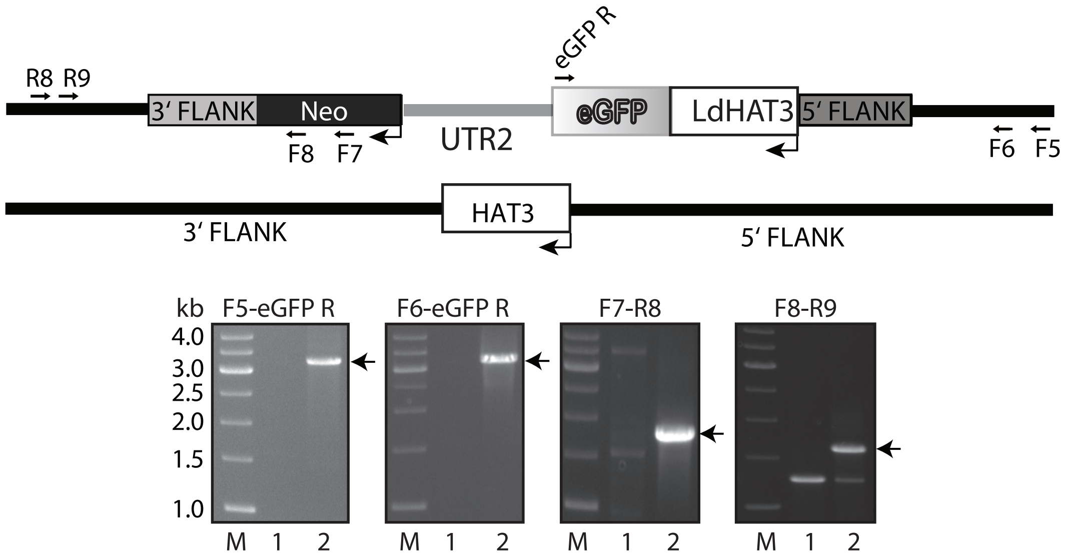
eGFP R
R8 R9
3' FLANK
Neo
eGFP
LdHAT3
5' FLANK
F8 F7
UTR2
F6 F5
HAT3
3' FLANK
5' FLANK
kb
F5-eGFP R
F6-eGFP R
F7-R8
F8-R9
4.0
3.0
2.5
2.0
1.5
1.0
M 1 2
M 1 2
M 1 2
M 1 2

B.

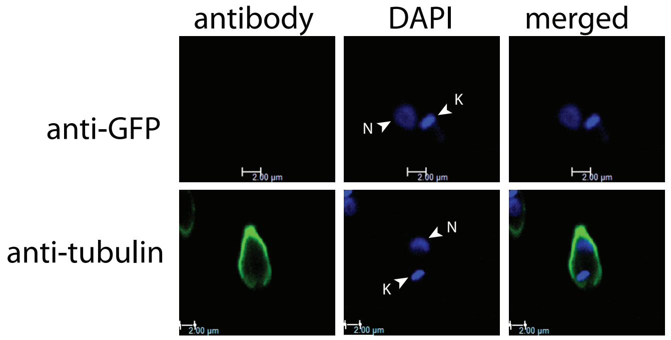
antibody
DAPI
merged
anti-GFP
anti-tubulin
N
K

Figure S2

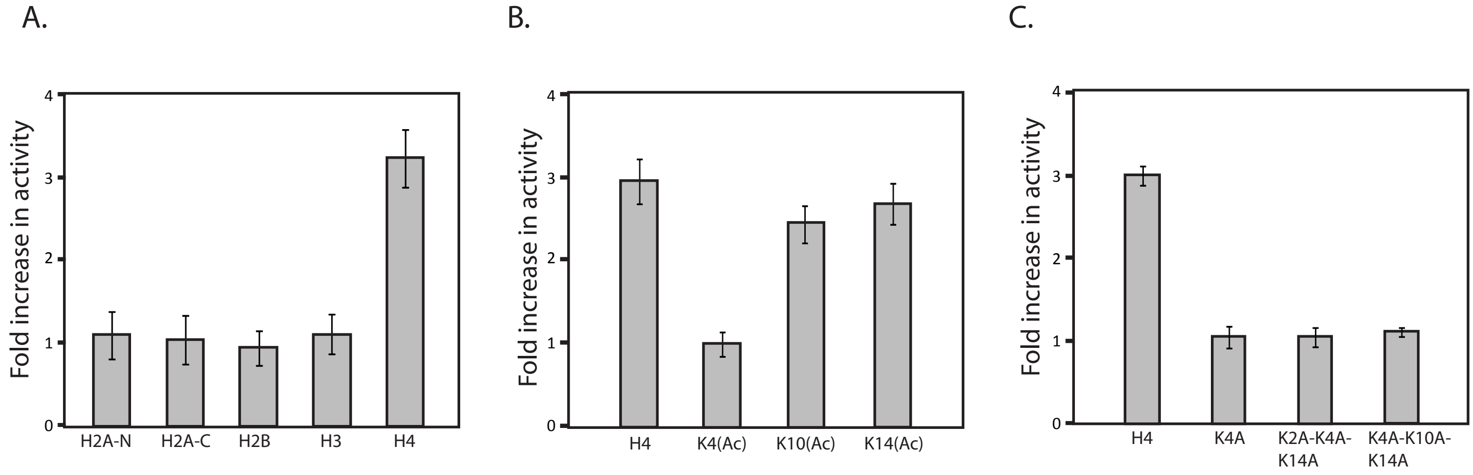

Figure S3

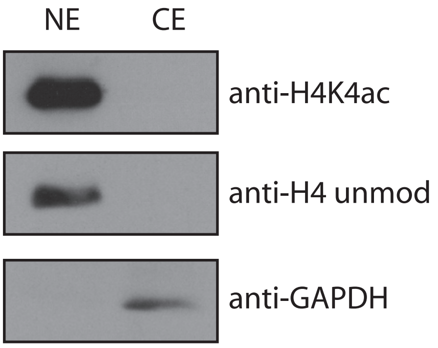

Figure S4

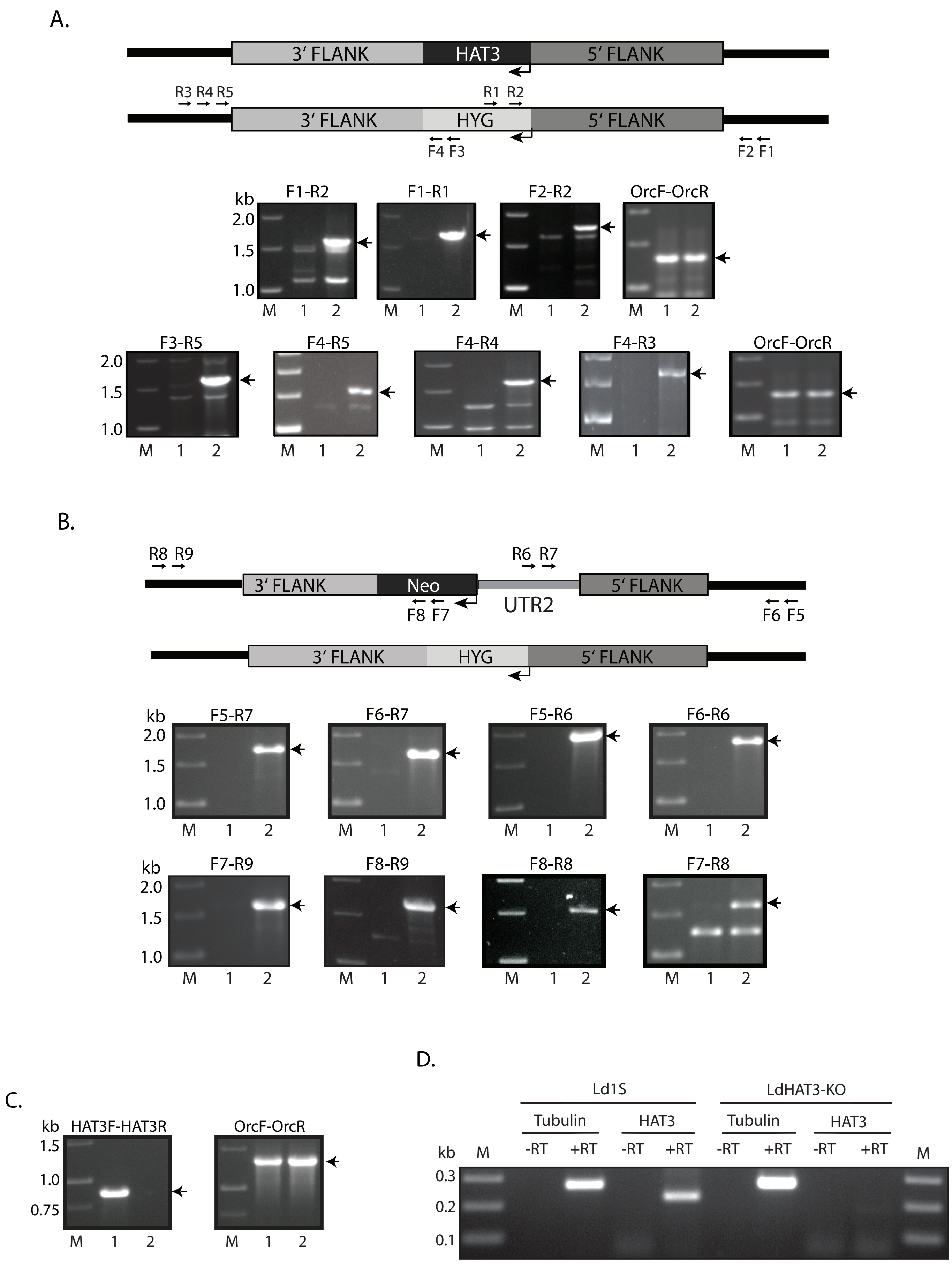

A.
3′ FLANK
HAT3
5′ FLANK
R3 R4 R5
R1 R2
HYG
F4 F3
F2 F1
F1-R2
F1-R1
F2-R2
OrcF-OrcR
kb
2.0
1.5
1.0
M 1 2
F3-R5
F4-R5
F4-R4
F4-R3
B.
R8 R9
R6 R7
Neo
UTR2
F8 F7
F6 F5
F5-R7
F6-R7
F5-R6
F6-R6
F7-R9
F8-R9
F8-R8
F7-R8
C.
HAT3F-HAT3R
0.75
D.
Ld1S
LdHAT3-KO
Tubulin
HAT3
-RT
+RT
0.3
0.2
0.1

Figure S5

A.

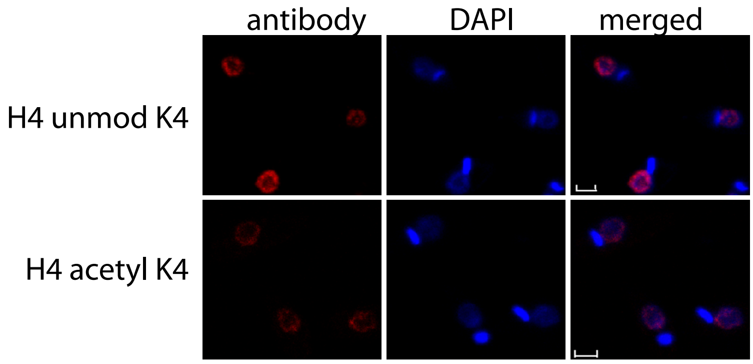
antibody
DAPI
merged
H4 unmod K4
H4 acetyl K4

B.

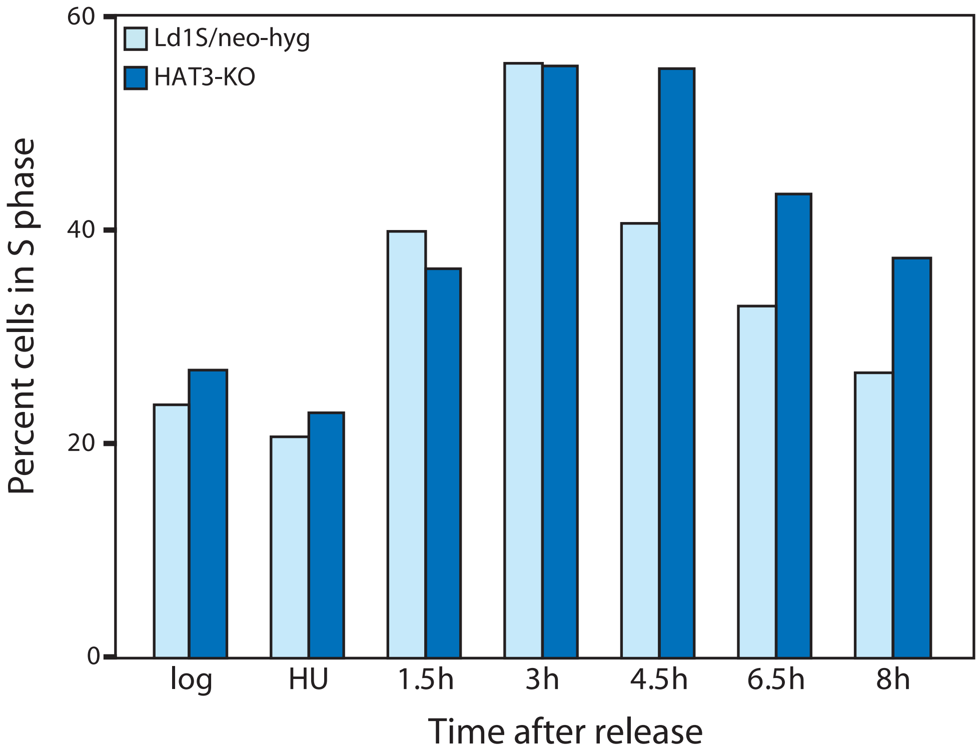
Ld1S/neo-hyg
HAT3-KO
Percent cells in S phase
60
40
20
0
log
HU
1.5h
3h
4.5h
6.5h
8h
Time after release
